# Predictive models of weakness among older adults: the contribution of oral health indicators

**DOI:** 10.1590/1807-3107bor-2025.vol39.064

**Published:** 2025-09-29

**Authors:** Ana Lúcia Schaefer Ferreira de MELLO, Mateus Cardoso PEREIRA, Daniela de Rossi FIGUEIREDO, Eleonora D’ORSI, Marco Aurélio PERES, Karen Glazer PERES

**Affiliations:** (a) Universidade Federal de Santa Catarina – UFSC, Graduate Program in Dentistry, Florianópolis, SC, Brazil.; (b) Universidade do Sul de Santa Catarina, Palhoça, SC, Brazil.; (c) Universidade Federal de Santa Catarina – UFSC, Graduate Program in Public Health, Florianópolis, SC, Brazil.; (d) National Dental Research Institute of Singapore, Health Services and Systems Research Program, Duke-NUS Medical School, Singapore.

**Keywords:** Frailty, Muscle Weakness, Oral Health, Forecasting

## Abstract

Poor oral health can negatively impact overall health and quality of life. Understanding how oral health predicts weakness in older adults is critical, since weakness increases the risk of health outcomes. However, the predictive role of oral health indicators in weakness among older adults remains unclear. This study assessed the ability of oral health indicators to predict weakness using data from Brazil’s EpiFloripa Aging cohort study. Predictive validity was evaluated in a sample of older adults participating in the cohort’s second (n = 440) and third (n = 347) waves. Self-reported sociodemographic, general health, and oral health variables were analyzed, with weakness diagnosed using cut-off points for handgrip strength. Predictive models incorporating sociodemographic, general health, and oral health variables were tested. Receiver operating characteristic curves, sensitivity and specificity, and positive and negative predictive values were calculated. Approximately 45.9% of the participants had two to three compromised oral health indicators during the second wave, and the five-year incidence of weakness was 31.9%. Oral health indicators and the oral frailty score did not enhance the prediction of weakness compared to models based solely on demographic, socioeconomic, and general health variables. However, models including oral health indicators demonstrated predictive accuracy comparable to those with demographic, socioeconomic, and general health variables. Sensitivity values were low (3.70-6.48%), while specificity values were high (>99%), with accuracy ranging from 0.64 to 0.71. These findings suggest that oral health indicators offer comparable predictive validity for weakness as sociodemographic and general health models, potentially serving as useful tools for health teams in screening older adults for weakness.

## Introduction

Aging is a complex process characterized by the decline of physical and mental capacities, reduced functionality, and the accumulation of diseases.^1,2^ These changes highlight the importance of investigating health conditions and their predictive factors in older adults to guide the development of effective health-oriented preventive care interventions.^2^

Handgrip strength (HGS) is a well-established marker of overall muscle strength and a valuable indicator of older adults’ health status.^3^ HGS measurements^4,5^ are widely used to assess muscle strength capacity and establish health-related inferences, including weakness cut-off points.^6^ Weakness, identified through HGS, is strongly associated with poor physical performance, mobility limitations^4^, frailty^7^, and sarcopenia in older adults.^8^ Moreover, HGS has predictive validity for cognitive decline, mobility impairments, functional status deterioration, and mortality in community-dwelling older adults.^3,9^

Given that poor oral health adversely affects general health and quality of life,^10-14^ further investigation into its relationship with HGS is warranted. Understanding oral health conditions may provide insights into factors influencing HGS and related outcomes. Despite this potential, data examining the predictive role of self-reported oral health conditions and oral frailty on HGS have not been made widely available. This study aims to assess whether self-reported oral health indicators can predict weakness in a cohort of older adults. Additionally, it seeks to evaluate whether combining oral and general health indicators can improve the accuracy of weakness prediction in older adults.

## Methods

### Study Setting and Design

The predictive validity of the oral health indicators was tested using data from the second and third waves of the EpiFloripa Aging cohort study,^15^ a prospective population-based cohort study comprised of residents aged 60 and older at baseline in Florianópolis, Santa Catarina, Brazil. According to the 2010 Census,^16^ Florianópolis had a population of 433,158, with 11.5% aged 60 or older. The city’s Municipal Human Development Index in 2010 was 0.847, ranking first among Brazilian capitals.^17^

### Sampling

The baseline wave of the EpiFloripa Aging cohort study was conducted in 2009/2010, with follow-ups in 2013/2014 and 2017/2018/2019. A total of 1,702 older adults participated in the baseline wave, 1,197 in the second wave,^18^ and 1,335 in the third wave.^19^ Detailed sampling and study procedures have been published previously.^15^ In the second wave, HGS measurements were obtained from 604 participants, with follow-up data available for 443 participants in the third wave. For this study, only data from the second and third waves were included, specifically from participants who did not present weakness in the second wave, thus allowing for the assessment of new cases of weakness over time.

### Outcome

Weakness was assessed by HGS evaluation, performed at participants’ homes by trained interviewers.^18^ HGS was measured in kilogram-force (kgf) using a dynamometer (Takei Kiki Kogyio® TK 1201, Japan) on the arm demonstrating greater strength.^3-5^ Weakness was defined based on established HGS cut-off points: (< 26 kgf for men and < 16 kgf for women).^6^This variable was analyzed dichotomously (weakness: yes/no).

### Predictors

Data were collected through face-to-face interviews using validated questionnaires. Responses were self-reported during the second wave.

Variables investigated:

- Edentulism (yes/no): Defined as self-reported loss of all upper and lower teeth.
- Self-rated oral health: Dichotomized into ‘good’ (great or good) and ‘poor’ (fair, bad or very bad), based on a five-point Likert scale.^20^
- Perceived need for treatment (yes/no): Assessed by the question, “Do you believe you need dental treatment?”
- Use and perceived need for complete dentures: Dichotomized into ‘yes’ or ’no’ for both upper and lower arches.
- Oral frailty: Measured using a score based on self-reported conditions, including edentulism, poor self-rated oral health, perceived need for treatment, current use of complete dentures, perceived need for full dentures, frequent xerostomia, and chewing difficulties. Scores ranged from 0 to 7 and were categorized into three groups: 0-1, 2-3, and 4-7 for further analysis.^12^
- Self-perceived health: Dichotomized into positive and negative^21^.
- Smoking status: Categorized as never smoked, former smoker, and current smoker^22^.
- Alcohol consumption: Dichotomized as yes or no.
- Functional dependence in activities of daily living ADL: Dichotomized as ‘yes’ for difficulty with four or more of 15 items^23^, and ‘no’ for fewer than four items.
- *Falls* reported in the last year (yes/no).
- Demographic and socioeconomic variables: These included sex, age (60-69, 70-79, 80+ years), marital status (married, single, divorced/separated, and widowed), education (no formal education; 1-4, 5-8, 9-11, or 12+ years of schooling), and income (measured in monthly minimum wages).^24,25^

### Data Analysis

Descriptive analyses were conducted to estimate the prevalence of all variables, with results presented as percentages and 95% confidence intervals (CIs). Multivariable logistic regression models were developed to predict weakness:

- Full Model: Included all independent variables.
- Reduced Model 1: Included variables with p-values <0.2 in the bivariate logistic regression.
- Reduced Model 2: Included variables with p-values <0.05 in the bivariate logistic regression.
- Akaike Information Criterion (AIC) Model: Selected based on AIC values for the best fit.

The discriminatory ability of each model was assessed by generating Receiver Operating Characteristic (ROC) curves with corresponding 95% CIs. Accuracy was categorized as follows: no discrimination (accuracy = 0.5), acceptable (0.7–0.8), good (0.8–0.9), and excellent (> 0.9).^26^ Sensitivity, specificity, positive predictive value (PPV), and negative predictive value (NPV) were calculated to describe model accuracy. Sensitivity and specificity values were classified as low (< 0.6), moderate (0.6–0.79), and high (> 0.8).^26^ Analyses were performed using Stata software version 13.0 (StataCorp, College Station, USA).

### Ethical Considerations

The EpiFloripa Aging cohort study was approved by the institutional ethics committee. All participants provided written informed consent.

## Results

**Figure 1:**
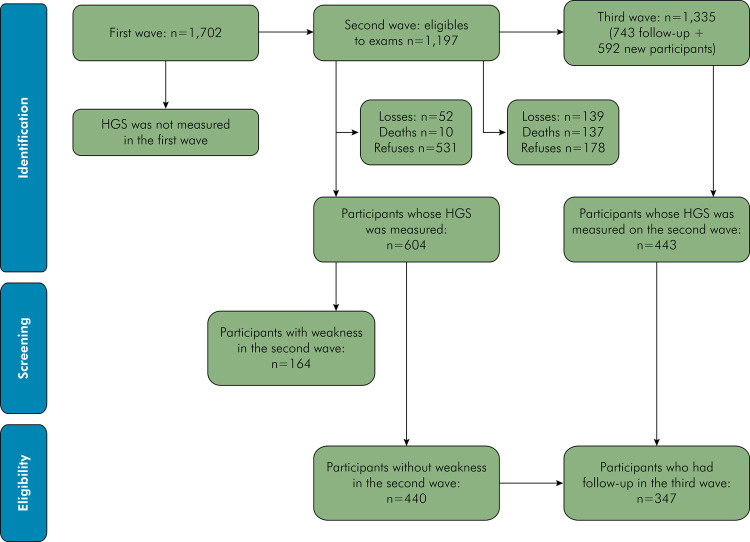
Study flowchart. HGS=Handgrip strength.

Data were analyzed from 440 participants without weakness in the second wave, and 347 were followed up in the third wave. Figure 1 presents the flowchart with the target population selection process. The participation rates were 50.8% in the second wave and 43.6% in the third wave, relative to the baseline/first wave.

Table 1 provides a descriptive analysis of the sample based on the independent variables and the outcome. Two to three oral frailty indicators were observed in 45.9% of the sample, while 16.9% had four or more indicators. The incidence of weakness over the 5-year follow-up period was 31.9% (n = 111).

**Table 1:** Description of the sample, including outcome (weakness) in the third wave (2017-2019), and self-reported demographic, socioeconomic, general health, and oral health variables from the second wave (2013-2014). EpiFloripa Aging cohort study, Florianópolis, Brazil.

Variable	n	%	95% CI
Handgrip strength (HGS) (n = 347)			
Normal	236	68.0	62.8–72.7
Reduced (weakness)	111	31.9	27.2–37.1
Sex (n = 440)			
Male	151	34.3	30.0–38.8
Female	289	65.6	61.1–69.9
Age (in years)(n = 440)			
60–69	215	48.8	44.1–53.5
70–79	181	41.1	36.6–45.8
≥ 80	44	10.0	7.5–13.1
Marital status (n = 440)			
Married	259	58.8	54.1–63.3
Single	29	6.5	4.6–9.3
Divorced or separated	38	8.6	6.3–11.6
Widowed	114	25.9	22.0–30.2
Education (in years) (n = 440)			
≥ 12 (university)	125	28.4	24.3–32.8
9– 11 (high school)	78	17.7	14.4–21.5
5– 8 (middle school)	77	17.5	14.2–21.3
1–4(primary school)	138	31.3	27.1–35.8
No formal schooling	22	5.0	3.3–7.4
Per capita income (in MW*) (n = 429)			
> 10**	27	6.29	4.3–9.0
4–10	85	19.81	16.2–23.8
2–4	97	22.61	18.8–26.8
0–2	220	51.28	46.5–56.0
Self-perceived health (n = 433)			
Positive	275	63.5	58.8–67.9
Negative	158	36.4	32.0–41.1
Smoking status (n = 440)			
Never	264	60.0	55.3–64.4
Former smoker	143	32.5	28.2–37.0
Current smoker	33	7.5	5.3–10.3
Alcohol consumption (n = 440)			
No	230	52.2	47.5–56.9
Yes	210	47.7	43.0–52.4
Functional dependence in ADL (n = 438)			
No	354	80.8	76.8–84.2
Yes	84	19.1	15.7–23.1
Falls reported in the last year (n = 440)			
No	321	72.9	68.5–76.9
Yes	119	27.0	23.0–31.4
Edentulism (n = 433)			
No	323	74.6	70.2–78.4
Yes	110	25.4	21.5–29.7
Self-reported oral health (n = 433)			
Good	284	65.5	60.9–69.9
Poor	149	34.4	30.0–39.0
Perceived need for treatment (n = 433)			
No	247	57.0	52.3–61.6
Yes	186	42.9	38.3–47.6
Use of complete dentures (n = 433)			
No	209	48.2	43.5–52.9
Yes	224	51.7	47.0–56.4
Perceived need for complete dentures (n = 433)			
No	294	67.9	63.3–72.1
Yes	139	32.1	27.8–36.6
Xerostomia (n = 433)			
Never	343	79.2	75.1–82.7
Often	90	20.7	17.2–24.8
Chewing difficulties (n = 433)			
No	391	90.3	87.1–92.7
Yes	42	9.7	7.2–12.8
Oral frailty (indicator) (n = 427)			
0 –1	156	36.5	32.0–41.2
2–3	188	44.0	39.3–48.7
4–7	83	19.4	15.9–23.4

ADL: Activities of daily living; HGS: Handgrip strength; CI: Confidence interval; MW: Minimum wages. *Reference value = Brazilian MW at the time of data collection. MW: R$724≅USD 133 in 2014).^26^ The responses were categorized according to the Brazilian criteria of social classes from the highest (>10 MW) to the lowest social class (0 to 2 MW);^27^ **Because of the low number of elderly people in the ‘> 20 MW’ category (n=3) and for the purpose of further analysis, this category was grouped with the previous category, which was ‘10 to 20 MW’ and then became ‘> 10 SM’.

Table 2 shows the predictive models incorporating demographic, socioeconomic, and general health variables. All three models demonstrated similar performance, characterized by low sensitivity (3.7%), high specificity (>99%), and moderate accuracy (0.69). These findings suggest that the models have strong potential for identifying individuals without weakness. However, their ability to predict weakness is limited, as indicated by a low PPV (0.1) and a relatively high NPV (0.89). These results indicate that the models are effective in predicting the absence of weakness, but less reliable for identifying its occurrence.

**Table 2:** Sensitivity, specificity, accuracy, negative predictive value (NPV), and positive predictive value (PPV) of predictive models of weakness with studied variables. EpiFloripa Aging cohort study, Florianópolis, Brazil.

Variable	Model	Threshold (%)	Sensitivity (%)	Specificity (%)	Accuracy	NPV	PPV
DEM, SE, and GH	Full	69.14	4.63	99.56	0.71	0.89	0.10
DEM, SE, and GH	Reduced*	68.93	3.70	99.57	0.69	0.89	0.10
DEM, SE, and GH	Reduced model**	68.93	3.70	99.57	0.69	0.89	0.10
DEM, SE, GH, and independent OH variables	Full model	69.73	6.48	99.56	0.71	0.90	0.10
DEM, SE, GH, and independent OH variables	Reduced model*	68.64	3.70	99.13	0.69	0.89	0.10
DEM, SE, GH, and independent OH variables	Reduced model**	69.23	4.63	99.57	0.69	0.89	0.10
DEM, SE, GH, and OF	Full model	68.84	3.70	99.56	0.71	0.89	0.10
ADEM, SE, GH, and OF	Reduced model*	68.93	3.70	99.57	0.69	0.89	0.10
DEM, SE, GH, and OF	Reduced model**	68.93	3.70	99.57	0.69	0.89	0.10

DEM: demographic variables; SE: socioeconomic variables; GH: general health variables; OF: oral frailty; OH: ral health. *Reduced Model 1 including variables with p-value < 0.2 by univariate logistic regression; **Reduced Model 2 including variables with p-value < 0.05 by univariate logistic regression.

Table 2 also presents the predictive models incorporating oral health variables alongside the demographic, socioeconomic, and general health variables. The full model demonstrated an improvement in sensitivity (6.48%) compared to the previous models, suggesting a slightly better ability to identify weak older adults. Specificity remained consistently high across all models (> 99%), highlighting the predictive strength in identifying non-weak individuals. Moderate accuracy remained relatively stable (0.69 to 0.71), while the NPV consistently held at 0.89, affirming the reliability of the models in predicting the absence of weakness. However, the PPV remained low (0.10) across all models. Incorporating the oral frailty indicator into the models (Table 2) yielded performance metrics similar to the previous models, indicating no significant improvement in sensitivity (3.7%) or accuracy metrics (0.69 to 0.71). Nonetheless, models with oral health variables achieved accuracy metrics comparable to those with general health variables, further demonstrating the predictive consistency of the models.

**Figure 2:**
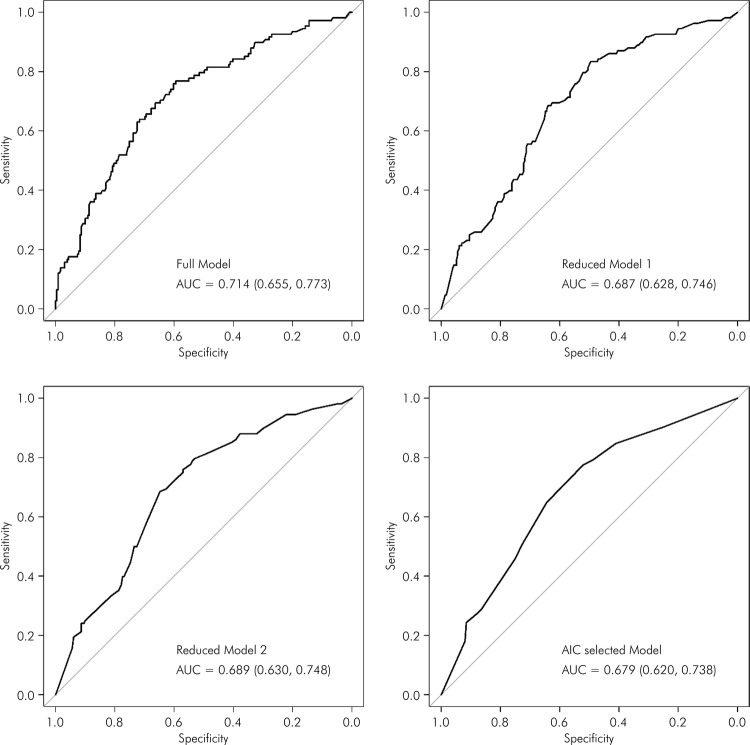
The ROC curves for demographic, socioeconomic and general health variables predicting weakness among older adults. EpiForipa Aging Cohort Study.

**Figure 3:**
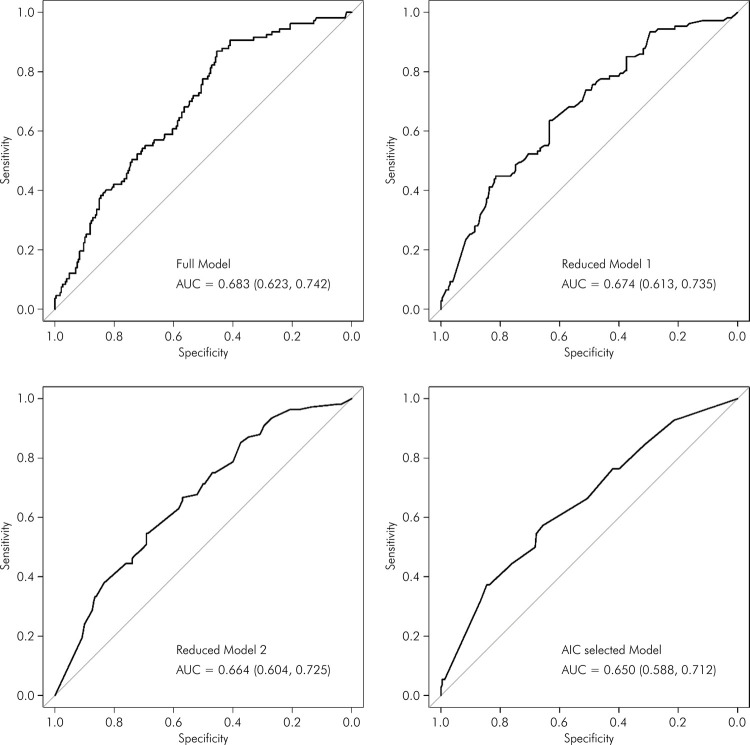
The ROC curves of demographic, socioeconomic, general health, and self-reported oral health variables for predicting weakness among older adults. EpiForipa Aging cohort Study.

**Figure 4:**
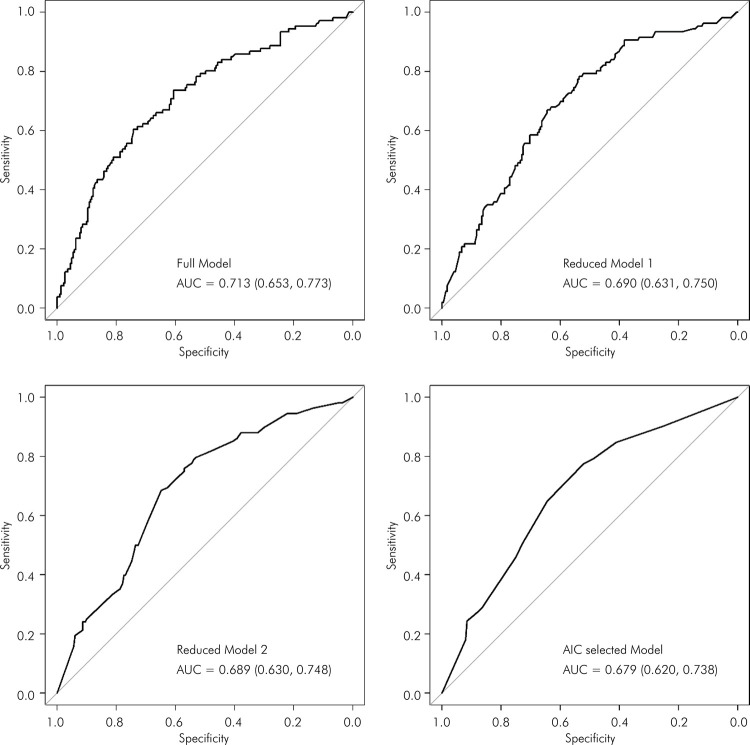
The ROC curves of demographic, socioeconomic, general health, and oral frailty variables for predicting weakness among older adults. EpiForipa Aging cohort Study.

Figures 2, 3, and 4 display the ROC) curves for the respective models. The AIC-selected models revealed areas under the curve (AUC) of 0.65 for the first set of variables, 0.679 for the second set, and 0.672 for the third set, indicating moderate discriminatory power.

## Discussion

This study evaluated the ability of oral health indicators, combined with general health indicators, to enhance the prediction of weakness in older adults. We tested predictive models incorporating individual oral health indicators and an oral frailty score. Our findings revealed that the self-reported oral health indicators did not improve the prediction of weakness in models already accounting for demographic, socioeconomic, and general health variables. However, these oral health indicators demonstrated predictive validity comparable to general health indicators.

The results showed low sensitivity across all models, indicating limited ability to identify the weak individuals correctly among the positive cases. Conversely, specificity was consistently high, highlighting the strong capacity of the models to classify non-weak older adults accurately among the negative cases. Accuracy remained stable across the models, ranging from 0.69 to 0.71, reflecting balanced predictive performance for both weak and non-weak individuals. The NPV remained stable at approximately 0.89, suggesting reliability in correctly predicting non-weak cases. However, the PPV was low at 0.10, indicating that predictions of weakness were frequently incorrect. The AIC analysis revealed AUC values ranging from 0.65 to 0.679,^8^ indicating the moderate discriminatory power of the models. This suggests that while the models possess some predictive ability, they are not effective in distinguishing between weak and non-weak cases with high precision.

The ROC analysis highlights the moderate discriminatory ability of these models to predict weakness. However, the low sensitivity and PPV values indicate that the three models detect weakness poorly. This limitation may result from the smaller number of weak compared to non-weak individuals, or the complexity of the underlying relationships between predictors and HGS, leading to reduced sensitivity rates. Furthermore, it suggests that individuals who self-report poor general health tend to report similarly for oral health, which may render these measures equivalent in the predictive model. On the other hand, the high specificity values demonstrate that the models effectively identify non-weak individuals among negative cases, minimizing the false-positive rate and enhancing their ability to rule out non-weak older adults.

The similarity in performance metrics between the full and the reduced models indicates that incorporating the oral frailty variable did not significantly improve sensitivity or other predictive measures. This suggests that oral frailty may not possess strong predictive power for identifying weaknesses, or may interact with other features in complex ways. Adverse oral health conditions are increasingly recognized as precursors to physical decline and frailty.^27,28^ The concept of oral frailty, defined as diminished oral function, highlights frailty manifesting specifically in the oral cavity.^29^ It is a valuable framework for developing oral health policies.^30^While several authors have explored its theoretical aspects and measurement approaches,^31^ standardization remains lacking.^28,32,33^ Nevertheless, common parameters include the number of teeth, saliva production, tongue condition, mouth pain, and oral function. Recent consensus emphasizes the need to assess oral functionality in older adults receiving care in primary healthcare settings.^34^

Regarding the accuracy analysis, models with and without oral health indicators demonstrated similar accuracy values (approximately 0.7), classified as acceptable.^26^ This suggests that the models consistently make accurate predictions for both weak and non-weak individuals. However, the inclusion of self-reported oral health indicators, whether individually or as part of an oral frailty score, did not significantly enhance the predictive validity of models already incorporating general variables for weakness. Notably, oral health indicators exhibited accuracy comparable to general health variables, implying their potential as substitutes for general health variables when predicting weakness in older adults, provided that the accuracy remains at similarly acceptable levels.

This study relied exclusively on self-reported oral health variables. While some studies support the validity of self-reported data for oral conditions, its reliability remains a subject of debate. Unlike clinical examinations, self-reported data collection requires fewer resources, less time, and no specialized examiners, thus enabling the generation of extensive data through a single interview. These attributes make self-reported data appealing for screening purposes, particularly in health surveillance contexts. However, further research is needed to evaluate the feasibility of using self-reported oral health conditions as a reliable screening tool.

Nearly one-third of the participants experienced a five-year incidence of weakness. In the study that established the cut-off points for weakness used herein, prevalence rates were 5% in men and 18% in women.^6^ While several studies have explored the predictive potential of HGS for adverse health conditions, fewer have investigated reduced weakness as an outcome of other conditions in older adults. One study identified age and hyperhydration as predictors of reduced HGS in both sexes, with serum magnesium levels predictive only in men.^35^However, HGS was analyzed as a continuous variable, and the accuracy of the predictive models was not measured, making direct comparisons with our findings challenging. HGS testing is recognized as a key factor in predicting sarcopenia, and has proven effective for identifying individuals who require further diagnostic tests to confirm sarcopenia.^28^ This raises the question of whether self-reported oral health indicators could serve as a practical screening tool for multi-professional health teams in routine weakness assessments for older adults.^36^

In the second wave of the EpiFloripa Aging cohort study, HGS was measured in 604 out of 1,197 participants, primarily due to a high refusal rate. Notably, HGS measurements were conducted in a university setting rather than participants’ homes^19^, and only those who could attend the university were included. This limitation may have introduced selection bias, potentially leading to an underestimation of weakness prevalence. In the third wave, HGS was measured at the participants’ homes, thus allowing for broader inclusion. If HGS continues to be assessed at home in future waves, predictive models for weakness could be developed and tested with larger sample sizes. However, the selection and survival biases remain critical concerns for generalizing results, since participant losses in the third wave accounted for 30% of the initial sample.

Our study underscores the importance of integrating oral health examinations into health assessments for older adult populations from a multi-professional perspective. Additionally, it highlights the need to enhance oral health screening tools to better predict the development of complex general health conditions, such as weakness. Geriatric dentistry plays a vital role in public health initiatives,^37^since oral health problems can significantly impact an individual’s quality of life and well-being.^13^Integrating oral health assessments of older adults into routine check-ups conducted by trained health professionals is a reasonable and actionable approach. Furthermore, incorporating HGS measurements as a routine examination could provide valuable insights into older adults’ health and status. The predictive value of HGS for disability, frailty, and mortality is well-documented,^38^ making HGS a reliable, low-cost, and early indicator of adverse health outcomes in public health settings.

Researchers and health professionals should recognize that model improvements may be necessary depending on the priority of accurately identifying weak older adults. The ROC results provide valuable insights into the strengths and limitations of predictive models for weakness. Interpreting these results requires evaluating the trade-offs between sensitivity and specificity, identifying potential limitations and factors influencing model performance, and exploring strategies to enhance prediction accuracy. Potential steps for improvement include incorporating new features that account for interactions between oral health variables to better capture their relationship with weakness, ensuring comprehensive and representative data for the target population, and identifying additional relevant predictors. A low predictability level highlights the need for developing new measures, alternative data collection methods, and innovative empirical approaches.^39^

This study is a pioneering exploration of oral frailty and weakness, with the cohort design being a notable strength, since it provides a temporal framework to generate robust scientific evidence.^40^ The EpiFloripa Aging cohort study represents a large and valuable dataset capable of revealing complex relationships that are challenging to hypothesize, particularly regarding emerging concepts like oral frailty. The predictive modeling analysis offers the opportunity to uncover potential causal mechanisms and generate new hypotheses.^39^ Furthermore, the cut-off points for weakness were determined non-arbitrarily, based on a large study of older adults, thus enhancing the reliability of the findings.^6^

Limitations must be acknowledged when interpreting these results and applying the models in real-world scenarios. Low sensitivity may have implications for patient care, since it could result in false negatives and potential underdiagnosis of weakness. The ROC analysis underscores the need for further investigation and refinement of predictive models.

Self-reported edentulism, while valuable, has limitations as an oral health indicator due to its endpoint nature, reflecting the cumulative effect of oral diseases and its high prevalence among older adults. Nevertheless, the choice of edentulism is supported by theoretical justification and practical advantages. Clinically, edentulism is straightforward to identify, and reflects a lifetime of oral health, as well as systemic and behavioral factors associated with general health. Additionally, edentulism directly affects nutritional status, contributing to sarcopenia—a central component of frailty—and is closely associated with psychosocial factors in the development of frailty in older adults. Using edentulism as a predictor enables targeted rehabilitative interventions aimed at improving masticatory function, enhancing nutritional status, and potentially reducing the risk of frailty. Moreover, other conditions, such as nutritional deficiencies and comorbidities, could improve predictive models as relevant factors in weakness and frailty. However, it is recognized that functional capacity limitations and falling episodes are tangible outcomes of comorbidities or nutritional deficiencies, emphasizing the importance of a comprehensive approach in model development and patient care.

It is essential to identify potential sources of bias, consider other relevant health and oral health variables and their measurement methods, and explore alternative modeling techniques to enhance sensitivity while maintaining acceptable levels of specificity and accuracy. Future research should integrate more comprehensive measures, including clinical and epidemiological indicators, to further improve the predictive capacity of models addressing weakness among older adults.

## Conclusion

This study examined whether self-reported oral health indicators, combined with general health conditions, could enhance the predictive ability of weakness, as determined by handgrip strength measures. The findings demonstrated that self-reported oral health indicators exhibited predictive validity for weakness similar to that of general health variables, supporting the importance of oral health screenings by health professionals. Such screenings may contribute to predicting the development of complex general health conditions, including weakness.

The accuracy analysis revealed low sensitivity and high specificity across all models. Self-reported oral health conditions, whether considered independently or grouped as oral frailty, did not significantly improve the prediction of weakness. Further research is needed to examine the predictive potential of adverse oral conditions, providing valuable insights and practical guidelines for health services aiming to address weakness in older adults.

## Data Availability

The authors declare that all data generated or analyzed during this study are included in this published article.
